# Bibliography

**Published:** 1897-09

**Authors:** 


### Bibliography.

THE AMERICAN TEXT-BOOK OF OPERATIVE DENTISTRY. IN CONTRIBUTIONS BY EMINENT AUTHORITIES. Edited by Edward C. Kirk, D.D.S., Professor of Clinical Dentistry in the University of Pennsylvania, Philadelphia. Editor of the *Dental Cosmos.* Illustrated with seven hundred and fifty-one engravings. Philadelphia and New York: Lea Brothers & Co., 1897.

A work on operative dentistry, covering all the modern phases of that branch of stomatology, has been for a long period a necessity, and it is a gratification that the liberality of the publishers, Lea Brothers & Co., has given to the dental profession, in the past year, two companion volumes, each, in its place, unequalled for careful and generally thorough treatment of the subjects supposed to specially belong to the two branches of practical dental work.

Both of these works have been given an encyclopædic character, a necessity where a number have written upon quite distinct subjects. This method of treating special branches of work has a good and a bad side. It loses, ordinarily, in that compact and steady advance from the simple to the complex, possible with the individual writer, and gains, or should gain, in a broader and more thorough consideration of subjects. While this is true, it still remains the fact that, after all, the matter resolves itself into individual opinions, resulting frequently in confusion through antagonistic ideas.

The present volume has overcome this, in a large degree, through very careful editing, and the editor, Dr. Kirk, is to be congratulated that he has been able to systematize this, and avoid the difficulty to a great extent. The amount of labor this has required cannot be appreciated except by those forced to do similar work; but it is certainly not the least of that given to the preparation of the volume.

The contributors are names well-known in dentistry,—Thompson, Andrews, Burchard, Case, Christensen, Clapp, Cryer, Darby, Goddard, Guilford, Jack, Ottofy, Peirce, and Thomas. These with the editor, Dr. E. C. Kirk, constitute a corps capable of doing justice to the subjects treated.

It would be unreasonable to expect that the views expressed will meet with universal concurrence, indeed, it may be expected that some of them will receive sharp criticism. The profession of dentistry is largely individualized, and this manifests itself in dogmatic assertion and very positive beliefs and more positive practice.

The book opens with a chapter on the "Macroscopic Anatomy of the Human Teeth," by Alton Howard Thompson, D.D.S. This is prepared in the writer's usual excellent style, and with that exact knowledge for which he is so widely known, and which is so universally appreciated.

"The Embryology and Histology of the Dental Tissues," by R. R. Andrews, A.M., D.D.S., F.R.M.S., is a masterly treatment of the subject, and very beautifully illustrated. The illustrations throughout the book are worthy of special notice, for these are not only, in the main, original productions, but they mark a dividing line between the old and the modern methods of preparing books of this character. Dr. Andrews gives the subject as it presents to the eye of the microscopist, and not, as formerly, drawings in which the personal equation largely prevails.

It is not the purpose of this review to follow individual contributors, for each has given, in his own way, his special thought and practice, but rather to follow the work as a whole, for it is in this success or failure must result either as a text-book or as a work of reference.

The difficulty has, doubtless, been experienced by the editor in defining exactly what is meant by operative dentistry. This term originated in the earlier work of the profession to distinguish the labor over the chair from that in the laboratory. The editor says, in his preface, "In determining the range of topics which may be properly classified as coming within the field of operative dentistry, the editor has been guided by the principle of distinguishing all those procedures the performance of which includes operative work upon the mouth as belonging to operative, and all those which are performed in the laboratory as pertaining to prosthetic dentistry." This would seem a reasonable definition, but as all the operations in the laboratory are merely preliminary to operations in the mouth, renders it difficult to make any sharp line of division. The result of this definition of the editor has been to exclude all crown- and bridge-work, or any of the forms of artificial substitutes, splints for fractures, or obturators. This is, doubtless, the correct course to pursue with this volume, as a companion to that previously issued, but, viewed as a distinct contribution to operative dentistry, it is open to criticism.

The principal object to be secured in a work of this kind is absolute simplicity of treatment. To effect this all subjects should, in the opinion of the reviewer, be treated upon the methods adopted by the true teacher, that of making everything clear to the dullest member of the class; the brilliant scholar can always take care of himself.

The shaping of cavities so that the beginner will find no difficulty in gaining support for his filling, as well as retaining the first piece of gold, is the most important of the entire operations of filling teeth. The mere packing of gold is a very trifling matter of mechanical skill. All teachers have found that the average student fails at the very first move, if he fails at all, and that is in not being able to recognize the necessity of a mechanical retention-place for the first piece of gold. The instructions upon the preparation of cavities, while in the main correct and lucid in explanation, fail, in the judgment of the reviewer, in not being clear upon this one vital point. *The student must be taught to anchor solidly his first piece of gold;* but the writer of this chapter does not, apparently, regard this of much importance, or, at least, does not specially dwell upon it.

The pathological relations of various operations in the mouth are of first importance, and cannot be separated from the mere mechanical side of the labor involved. In some of the chapters this is carefully looked after, in others, not to the extent of training the beginner to processes essential to his own success or the good of his patient. This defect is noticeable in the chapter on the rubber dam. There is, probably, no appliance that has caused more gingivitis than this valuable aid to dental operations. In itself it is, in degree, harmless; but when the accessories are placed in position, clamps and ligatures, the result is irritation and frequently severe inflammation at the gingival border after a prolonged operation. Now, this means, when the dam is removed, a wounded gum tissue, and frequently an irritated pericementum. It is a truism in pathology to assert that with this condition in the mouth there is established an excellent culture medium for pathogenic germs, and they are never slow to take advantage of the opportunity. In fact, in careless hands, in forty-eight hours their development will be manifested, where injury has resulted, as described, in acute pericementitis or the incipient stages of pyorrhœa. It seems to the reviewer that it would have been well to raise the danger signal here and have given good advice as to supplementary antiseptic measures to prevent pathological sequelæ. These criticisms apply with equal force to the use of the wedge. This is very useful in filling, properly used, but a very dangerous one in inexperienced hands, and this, it would seem, should have been stated.

Would it not have been better in writing, "That all metals become more or less stiff or rigid by hammering, but become soft again by the application of considerable heat," to give the reason for this change? The mere statement of fact does not carry with it the intelligence needed to be absorbed by the student. An error of not much moment is noticed in describing the placing of the dam where decay has extended beneath the gum margin, and a Mack screw is recommended "to be inserted two or three threads into *the dentine*." It is presumed the writer meant the cementum, for two or three threads of those fine screws would fail to penetrate the dentine at the cervical border.

With the exception of this allusion to the use of the screw, the reviewer fails to find any mention of these valuable aids in cavities of exceptional difficulty. Surely screws have not been relegated to the museum of antiquated appliances? In the opinion of the reviewer they not only retain a place, but a very valuable place, in operative dentistry, and should have been given their proper value in a work of this kind.

In the article on devitalization of the pulp, the author fails to make clear the importance of a previous condition of active congestion of the pulp prior to the use of arsenic. While recognizing this as antagonistic to the action of the agent, he seems not to make the fact clear that it is possible to destroy a pulp, even when actively congested, by the use of proper anæsthetics in connection with the devitalizing agent, and recommends as a primary operation the reduction of "the state of hyperæsthesia of the pulp and to relieve the congestion . . . before commencing the devitalization." This is the old method, but by the use of iodoform or cocaine this has been rendered unnecessary. In this connection it is stated that "iodoform has been much used in combination with arsenous acid in the devitalization of the pulp; its value depends upon its *disinfecting power*." As the reviewer was the originator of the idea of the use of iodoform in combination with arsenous oxide, it can be stated that its introduction was not based on its *antiseptic* property, which is almost valueless, but upon its *anæsthetic effect*, recognized the world over as being superior to any other agent, not excepting chloroform, and having the advantage over the latter that its action is continuous. Its use in combination with arsenous oxide was recommended because of this property of continually acting as a powerful sedative, lowering the tone of the local nerves, thus limiting the blood-current and reducing the hyperæsthetic state, and enabling the arsenic to enter the tissue by imbibition, and have its legitimate effect in paralyzing the nerve-supply and cutting off nutrition. This, when applied according to directions originally given, resulted in a painless devitalization of congested pulps. The same, in degree, occurs with cocaine. It is true, the author has given a formula of arsenous acid and cocaine, but it does not seem to have been used for the purpose described.

There does not appear to have been any attempt made to regulate the amount of arsenic to be used. This is impossible with the formula given or by the old one of Dr. J. D. White, of arsenic, morphine, and creosote. The teaching of the present time is to avoid all such combinations and to use the ingredients in exact proportions at the time these may be required. The old method is certainly empirical, as any definite quantity is out of the question, and this is of vital importance in dealing with an agent as destructive as arsenic. It seems that the student should be taught the nearest approximate amount of arsenic required to devitalize the pulp to the *upper third* of a single-rooted tooth. This understood, increased amounts can be applied in the trifurcated teeth with intelligence.

It would have been well to have thrown out a caution as to its use and the danger of producing serious sloughing, and the means to be resorted to in case arsenic should inadvertently be taken up by the gum tissue, an accident by no means uncommon, especially with beginners.

The value of the "diagrammatic representation of the serial decomposition of an infected pulp," on page 320, may be seriously questioned. To be of any value these imaginary drawings should be based on repeated observations, and even then they may be misleading. It is very doubtful whether the diagram in question gives a correct idea of the progress of destruction of the pulp. Indeed, it is not in accord with the recognized progress of inflammation, as the place of formation of pus is given at the apical foramen, when, pathologically considered, pus should have no specially defined limitations in an inflamed pulp, but should be and is invariably found distributed in various parts of that issue. It is not thought that this adds materially to this otherwise carefully prepared chapter.

The assertion that Maynard was the first to fill canals needs qualification. He has been generally credited with this, but there is evidence to prove that Hudson filled pulp-canals long anterior to the time of Maynard.

The treatment given for pericementitis is by no means satisfactory. There is no proper division made between pericementitis, temporary or chronic, or where it shades off into the acute form, and some of the treatment seems to the reviewer impracticable, if not injurious. The writer of the chapter is recommended to study up Arkoöys's classification of this pathological condition, for while he may not learn much in treatment, it will give ideas as to the systematic arrangement for study or for teaching. In the interest of truth, it must be said of this portion of the work that it fails in the most important essential of treatment, practical value, inasmuch as some of the proposed methods would tend to increase rather than palliate the symptoms.

It is difficult to understand this quotation from the "Clinical History of Acute Alveolar Abscess:" "In from twenty-four to forty-eight hours a spot of fluctuation makes its appearance at the summit of the swelling; the spot becomes yellow and soon opens, affording escape to the abscess contents." The progress of abscess varies with the class of teeth affected and the density of the alveolus, the duration ranging from twenty-four hours in the lateral incisors to a week in the inferior molars, but no hard-and-fast limitations can ever be made in the period of duration of this pathological condition.

The chapter on "Pyorrhœa Alveolaris" is well prepared, and is an excellent statement of the views held by the author, but will, probably, not be accepted by many readers as the final word upon the subject. Much space is given to the views entertained by those who regard pyorrhœa as "directly dependent on the deposition of the uric acid, urates, and calcium salts in the pericemental membrane," and of those the author furnishes the best and most brilliant example. It seems to the reviewer that, while a brief allusion to these views was certainly necessary and desirable, it would have been better to curtail theory, and in its place give more space to practical methods of treatment.

The chapter on "Discolored Teeth and their Treatment," by the editor, is a marked evidence of the intelligent growth of processes for overcoming this difficulty in practice. For more than twenty years after the writer of this review introduced his method of bleaching it was met by a coldness bordering on contempt, but now bleaching is regarded rightfully as being one of the most important procedures in operative dentistry. While the more recent methods have a value, possibly, far above those first introduced, the important fact is emphasized in this chapter that teeth by all the processes described have been bleached, and teeth rendered presentable that were, under the old let-alone policy, a continued disfigurement and a disgrace to dentistry as a profession. This chapter must be regarded as of great practical value.

The chapter on "Extraction of Teeth" is a well-prepared article, and profusely and intelligently illustrated, in this respect far in advance of any within the knowledge of the writer.

The practical side of the "Extraction of Teeth under Nitrous Oxide Anæsthesia" is given by a well-known specialist, and is therefore the product of long, practical experience, and must be of special value to students.

Space does not permit the reviewer to follow the remaining chapters with anything like a critical examination. These are "Local Anæsthetics and Tooth Extraction," "Plantation of Teeth," "Management of the Deciduous Teeth," "Orthodontia Exclusively as an Operative Procedure," and "The Development of Æsthetic Facial Contours." All of these are treated exhaustively and with the recognized ability of the authors.

The exceptions taken to some things in this volume are not made in a spirit of hypercriticism, but with the hope that future editions will correct defects, and that, in time, the dental profession will have a purely practical work on operative dentistry, eliminating as much as possible personal predilections. This book has less of this than might have been expected, but there is room for improvement. There has been a notable effort to make it a volume worthy of the confidence of the dental profession, and whether the general judgment will recognize it as authority or simply as a book of reference, it must be regarded as the best attempt made in this country or elsewhere to originate a work on operative dentistry worthy of the era in which it is published.

The book is prepared with the same excellent attention to details that marked the work on "Prosthetic Dentistry," recently reviewed in this journal. The issuing of two books in one year, with every effort on the part of the publishers to present these in the most satisfactory manner, is certainly worthy of special notice, and marks an epoch in dental literature second only to the publishing of the great work, "The American System of Dentistry," by the same house. These books should make part of the library of every dentist whose aim is to stand with the advanced workers in his calling.

